# Bacterial profile, antibiotic susceptibility pattern and associated risk factors of urinary tract infection among clinically suspected children attending at Felege-Hiwot comprehensive and specialized hospital, Northwest Ethiopia. A prospective study

**DOI:** 10.1186/s12879-020-05402-y

**Published:** 2020-09-16

**Authors:** Adugna Fenta, Mulat Dagnew, Setegn Eshetie, Teshome Belachew

**Affiliations:** 1 Wogera primary hospital, North Gondar, Gondar, Ethiopia; 2 College of Medicine and Health Sciences, School of Biomedical and Laboratory Sciences, Department of Medical Microbiology, University of Gondar, Gondar, Ethiopia

**Keywords:** Bacterial isolates, Antimicrobial susceptibility, Children, Associated factors

## Abstract

**Background:**

Urinary tract infection is one of the most common bacterial infections in children. Understanding the characteristics of uropathogens and their antimicrobial susceptibility pattern in a particular setting can provide evidence for the appropriate management of cases. This study aimed to assess the bacterial profile of urinary tract infection, their antimicrobial susceptibility pattern and associated factors among clinically suspected children attending at Felege-Hiwot Comprehensive Specialized Hospital, Northwest Ethiopia.

**Methods:**

A hospital-based cross-sectional study was conducted from February–April, 2019. A systematic sampling technique was employed. A mid-stream urine sample was inoculated on cystine lactose electrolyte deficient media and incubated for 24–48 h. Sub-culturing was done on Mac-Conkey and blood agar. Antimicrobial susceptibility test was done on Muller-Hinton agar. A binary logistic regression model was used to see the association between dependent and independent factors. A *p*-value< 0.05 at 95% CI was considered as statistically significant.

**Results:**

The overall prevalence of urinary tract infection was 16.7% (95% CI 12.4–21.1). Both Gram-negative and Gram-positive bacterial isolates were recovered with a rate of 44/50 (88%) and 6/50 (12%) respectively. Among Gram-negative isolates, *E. coli* 28/44(63.6%) was predominant while *S. saprophyticus* 2/6(33.3%) was prevalent among Gram-positive bacterial isolates. Overall, a high level of resistance to ampicillin, augmentin, and tetracycline was shown by Gram-negative bacteria with a rate of 44/44(100%), 39/44(88.6%), and36/44 (81.8%) respectively. About 33/50(66%) of overall multidrug resistance was observed (95% CI 52–78). About six Gram-negative bacterial isolates were extended spectrum beta-lactamase (ESBL) producers. Having a history of urinary tract infection (P-0.003, AOR 1.86–22.15) and male uncircumcision (p-0.00, AOR 5.5–65.35) were the independent variables that associate for urinary tract infections.

**Conclusion:**

In the present study, the prevalence of urinary tract infection among children was high and considerably a high proportion of multidrug resistance was observed. This result will have a significant impact on the selection of appropriate antimicrobial agents for the treatment of urinary tract infection.

## Background

Urinary tract infection (UTI) is a serious bacterial infection causing illness in infants and children. It is one of the most common bacterial infections faced by clinicians working in the developing world [1]. The term UTI is applied to a variety of clinical conditions ranging from the asymptomatic presence of bacteria in the urine to severe infection of the kidney with the development of sepsis. If poorly treated or undiagnosed, it is an important cause of long-term morbidities like; hypertension, failure to thrive, and finally go to end-stage renal dysfunction [2, 3]. Developing kidneys of infants and young children are more susceptible to damage from pyelonephritis [4].

The epidemiology of UTI during childhood varies by age, gender, circumcision status, and other factors. Boys are more susceptible during the first year of life, mostly in the first 3 months, among boys, uncircumcised infants have an eightfold higher risk; due to phimosis, limited retraction of the foreskin is significantly associated with an increase in UTIs in male infants [5], afterward, the incidence is mainly higher in girls, due to differences in anatomy [6]. Common symptoms of UTIs include burning sensation during urination, loss of bladder control, increased frequency of urination especially in small amounts, low back pain, cloudy, and bloody or foul-smelling urine [7]. About 5% of girls and 2% of boys experience at least one incident of UTI up to the age of 7 years [8, 9].

Urinary tract infection is among the most prevailing infectious diseases with a considerable financial burden on society. It affects approximately 150 million people worldwide per year. Populations that have a major risk of acquiring a UTI are newborns, preschool children, sexually active women, and older individuals of both sexes [10]. Studies from developing countries showed that around 10% of children with febrile illnesses have a UTI and it extends to 8–35% if the patient is malnourished children [3, 11].

It is estimated that globally 26% of deaths are due to infectious diseases such as UTIs of which 98% occur in low-income countries [7]. The prevalence of UTI in pediatrics of Asian countries was high. In Nepal, two studies were studied at different times from different areas and showed that 15.88 and 57% [12, 13] of prevalence was documented. Another study in India also showed a 48% prevalence [14].

In South African children, a hospital-based study on prevalence, spectrum, and impact of healthcare-associated infection showed that UTI takes an 11% share [15]. Similarly, the study of the prevalence of UTI in one Nigerian teaching hospital to determine the incidence of urinary tract infection among children and adolescents was 11.9% [16]. In Tanzania, Children who presented prolonged duration of fever (7 days or longer) were more likely to have UTI, 16.8% [17]. Another study in Kenya on the contribution of urinary tract infection to the burden of febrile illnesses in young children reported about 11.9% share [11].

In Ethiopia, different studies done in different study groups. The findings at Hawassa referral hospital among pediatric patients showed that the prevalence of UTI was 27.5% [8]. Significant level of UTI was also reported at Yekatit 12 hospital Addis Ababa, 15.9% [18] and at Gondar University hospital, 26.45% [19]. But, in the study area, only one study was done in 2013 by Yerega B et al., but authors couldn’t identify and test too many associated factors of UTI among children. Besides, antimicrobial resistance is very dynamic so that periodic regular screening is essential. Therefore, our study will help physicians to update their knowledge on prevalence, antimicrobial susceptibility pattern and associated risk factors to make informed diagnostic and therapeutic decisions in treating children.

## Methods

### Study setting, design, population, and sampling techniques

An institution-based cross-sectional study was done among UTI suspected children from February–April, 2019 at Felege-Hiwot Comprehensive Specialized Hospital, Bahir-Dar, Amhara regional state. Bahir-Dar is the capital of the Amhara regional state in Northwest Ethiopia. The Town is located 576 km from the capital city of the country, Addis Ababa. Based on the 2007 Census conducted by the Central Statistical Agency (Ethiopia), Bahir Dar town has a total population of 221,990. The hospital is a tertiary care referral hospital with around 400 beds and 9 operating tables serving over 7 million people. The hospital provides obstetrics, pediatric, internal medicine, ophthalmology, gynecology, and orthopedic surgery services [20]. The sample size (299) was determined by using a single population proportion formula by considering the prevalence of 26.45% [19], with a 95% confidence interval, and a 5% margin of error. Study participants were recruited by convenient sampling technique. Children who were ≤ 15 years and suspected of urinary tract infection were included in this study. However, children who took antibiotics 2 weeks before data collection and children who were critically ill were excluded. In this study, all patients visiting Felege-Hiwot Comprehensive and Specialized Hospital for a certain clinical disease diagnosis were considered as a source population. Whereas, children who are visiting Mother and Child Health (MCH) clinic for UTI diagnosis during the study period were considered as a target population.

### Data collection methods

The standard questionnaire was prepared from reviewed literature and pre-tested. It was prepared in English and translated to the local language (Amharic) then translated back into English to check the accuracy of the translation. The questionnaire design included socio-demographic characteristics and associated risk factors. Data collection was done after obtaining written informed consent from parents/guardians. Socio-demographic and data related to associate factors were collected with face to face interview.

### Urine sample collection

Clean voided mid-stream urine (MSU) specimens were collected from UTI suspected children in sterile bottles by the patient’s parent with the assistant of nurse and investigator and transported to the laboratory within 2 h of collection. Contamination was managed by giving proper instruction on how to collect the sample correctly. When the patient is unable to provide urine sample catheters were used [21].

### Culture and identification techniques

Collected urine from each patient was inoculated onto cysteine-lactose-electrolyte deficient agar CLED/ (Oxoid, Basingstoke, Hampshire, England) plates using a calibrated inoculating loop with a capacity of 0.001 ml. The inoculated plates were incubated for 24–48 h at 37 °C aerobically. If growth observed, Plates with a colony count of ≥10^5^cfu/ml were considered significant bacteriuria [22]. Then sub-cultured to Mac-Conkey agar (Oxoid, Basingstoke, Hampshire, England) and 5% sheep blood agar (Oxoid, Basingstoke, Hampshire, England) [22]. Bacterial isolates were characterized/identified by Gram stain and biochemical tests; i.e. for Gram-positive bacteria: catalase test, novobiocin disk test and coagulase test were done while for Gram-negatives: Triple sugar iron agar test, indole motility test, citrate agar test, lysine decarboxylase agar test, urea agar test and oxidase test were done.

### Antimicrobial susceptibility testing (AST)

Antimicrobial susceptibility test was carried out using the Kirby-Bauer disc diffusion method as per the Clinical Laboratory Standards Institute (CLSI) guidelines on Muller Hinton agar [23]. The suspension of 3–5 colonies of freshly grown test organism was prepared equivalent to 0.5 McFarland standards. The surface of the Muller-Hinton agar was then completely covered by rotating the swab with the suspension. The plates were allowed to dry for 3–5 min: then discs were evenly distributed on the inoculated plate using sterile forceps and incubated at 37 °C for 18–24 h. The diameter of the zone of inhibition around the disc was measured using a ruler. Results were interpreted as Sensitive, Intermediate, and Resistance based on CLSI 2018 guideline [23]. For statistical analysis purpose, “intermediate” sensitivity results of bacterial isolates was grouped to “resistant” sensitivity results. The following routinely used antimicrobials were tested: ampicillin [10 μg], gentamycin [10 μg], amox-clav [20/10 μg], cefoxitin [30 μg], cefotaxime [30 μg], ciprofloxacin [5 μg]; meropenem [10 μg], cotrimoxazole [trimethoprim, 1.25/sulfamethoxazole, 23.75 μg], ceftazidime [30 μg], chloramphenicol [30 μg], tetracycline [30 μg], nitrofurantoin [300 μg], and erythromycin [15 μg] [23]. Multi-drug resistance was defined as resistance of an isolate to three or more antimicrobial classes tested [24].

### Extended Spectrum β-lactamase detection

#### Screening to ESBL production test

Based on CLSI-2018, bacterial isolates showing zone of inhibition diameters ≤22 mm to ceftazidime [30 μg] or ≤ 27 mm to cefotaxime [30 μg] were subjected to ESBL production test [23]. Phenotypic confirmation of ESBL production was done by using the double-disk diffusion method; cefotaxime [30 μg] and cefotaxime-clavulanic acid [30/10 μg] or ceftazidime [30 μg] and ceftazidime-clavulanic acid [30/10 μg]. The bacterial suspension was prepared while taking 2–3 fresh colonies and adjusted to 0.5 McFarland standard. Lawn culture was done on the Mueller-Hinton Agar (MHA) plate. The ceftazidime and ceftazidime-clavulanic acid discs were placed at 20 mm apart on the agar surface and incubated overnight at 37 °C. After overnight incubation a ≥ 5 mm increase in zone diameter for either cefotaxime or ceftazidime tested in combination with clavulanic acid, were taken as indicative for ESBL production. *E. coli* ATCC 25922 was used as an ESBL-negative whereas *K. pneumoniae* ATCC 700603 was used as an ESBL-positive reference strain [23].

### Data processing and analysis

Data entry and analysis were done by using SPSS version 20 software. The results were presented through texts, tables, and graphs. Descriptive statistics were used to summarize socio-demographic data, bacterial profile and susceptibility patterns of isolates. Bivariate and multivariate logistic regression analysis was carried out to identify potential factors of urinary tract infection of children. A *P* value < 0.2 was considered statistically significant for bivariate analysis. Adjusted odds ratio at 95% CI was used to measure the association between potential risk factors and UTI in children. A *p*-value < 0.05 at 95% CI was considered as statistically significant.

### Ethical consideration

Ethical clearance was obtained from the University of Gondar Biomedical Laboratory Sciences, Ethical Review Committee. The reference number of the ethical letter was “Ref no SBMLS/2123/11”. Written parental consent/assent was obtained from parents or guardians of children after explaining the purpose and objective of the study. Study participants who were not willing to participate in the study would not be forced to participate. They were informed that all data and sample obtained from them were kept confidential by using codes instead of any personal identifiers and is meant only for the purpose of the study. Positive results were communicated to health care providers.

## Results

### Socio-demographic characteristics

A total of 299 urinary tract infections (UTI) suspected children were included in the present study. Of the total study subjects, 165 (55.2%) were males. The age range of the participants was between day 1 and 15 years with a median age of 6 years. About 158(53%) of study participants were in the age group of 6–15 years (Table 1).

**Table 1:** Socio-demographic characteristics of UTI suspected children attending at Felege- Hiwot Comprehensive Specialized Hospital (FHCSH), Northwest, Ethiopia, February–April, 2019

Socio-demographic characteristics	Frequency	Percentage
**Sex**		
Male	165	55.2
Female	134	44.8
**Age (in years)**		
< 1	36	12.0
1–5	105	35.2
6–15	158	52.8
**Residence**		
Urban	91	30.4
Rural	208	69.6
**Educational status of father**		
Unable to read and write	83	27.8
Primary	91	30.4
High school	64	21.4
College and above	61	20.4
**Educational status of mother**		
Unable to read and write	127	42.5
Primary	65	21.7
High school	58	19.4
College and above	49	16.4
**Total**	299	100

### Prevalence of bacterial isolates from UTI suspected children

The overall prevalence of urinary tract infections among children was 16.7% (95%CI: 12.4–21.1, *N* = 50). Of the total culture-positive participants, the majority, 30/50 (60%), were females (Table 1).

Both Gram-negative and Gram-positive bacterial species were recovered, 44/50 (88%) and 6/50(12%), respectively. Among Gram-negative bacterial species, *E. coli* were the most frequently isolated bacteria followed by *Klebsiella* species, *Citrobacter* species, 28/44 (63.6%), 7/44(15.9%), and 6/44(13.6%), respectively (Table 2). *S. saprophyticus* and *S. aureus* were Gram-positive bacteria isolated with a rate of 3/6 (50%) each (Table 3).

**Table 2:** Drug resistance patterns of Gram-negative bacterial isolates from urine samples of children at the FHCSH, Northwest, Ethiopia, February–April, 2019

Bacterial Iso.	Sen.	AMP	GEN	AMC	CTX	CTX	CIP	MER	COT	CAZ	CHL	TET	NIT
***E. coli*** **(*n*** **= 28)**	S	–	13 (46.4)	5 (17.9)	24(85.7)	23(82.1)	21 (75)	27(96.4)	12(42.9)	21 (75)	23(82.1)	6 (21.4)	23(82.1)
	I	–	7 (25)	7 (25)	1 (3.6)	1 (3.6)	1 (3.6)	–	4(14.2)	3 (10.7)	1 (3.6)	6 (21.4)	1(3.6)
	R	28(100	8 (28.6)	16(57.1)	3 (10.7)	4 (14.3)	6 (21.4)	1 (3.6)	12(42.9	4 (14.3)	4(14.3)	16(57.2)	4(14.3)
***K.pneumoniae*** **(*n*** **= 5)**	S	–	1 (20)	–	3 (60)	4 (70)	4 (70)	5 (100)	2 (40)	4 (80)	3 (60)	1 (20)	4 (80)
	I	–	2 (40)	–	2 (40)	–	–	–	–	–	–	–	1 (20)
	R	5 (100)	2 (40)	5 (100)	–	1 (20)	1 (20)	–	3 (60)	1 (20)	2 (40)	4 (80)	–
***K.ozaniae (n = 1)***	S		1 (100)	–	1 (100)	1 (100)	1 (100)	1 (100)	–	1 (100)	1 (100)	–	1 (100)
	R	1 (100)	–	1 (100)	–	–	–	–	1 (100)	–	–	1 (100)	–
***K.rhinoscleromatis*** **(*n*** **= 1)**	S	–	–	–	1 (100)	1 (100)	–	1 (100)	1 (100)	1 (100)	1 (100)	–	1 (100)
	I	–	–	1 (100)	–	–	1 (100)	–	–	–	–	–	–
	R	1 (100)	1 (100)	–	–	–	–	–	–	–	–	1 (100)	–
***P.vulgaris*** **(*n*** **= 1)**	S	1 (100)	1 (100)	–	1 (100)	1 (100)	1 (100)	1 (100)	1 (100)	1 (100)	1 (100)	1 (100)	1 (100)
	R	–	–	1 (100)	–	–	–	–	–	–	–	–	–
***Citrobacter*** **spps. (*n*** **= 6)**	*S*	–	3 (50)	1 (16.7)	1 (16.7)	1 (16.7)	5 (83.3)	6 (100)	1 (16.7)	2 (33.33)	1(16.7)	–	3 (50)
	I	–	2 (33.3)	–	–	–	1 (16.7)	–	–	2 (33.33)	1(16.7)	1 (16.7)	–
	R	6 (100)	1 (16.7)	5 (83.3)	5 (83.3)	5 (83.3)	–	–	5 (83.3)	2 (33.33)	4(66.7)	5 (83.3)	3 (50)
***Providencia spps*. (*n*** **= 1*)***	S	–	1 (100)	–	1 (100)	1 (100)	1 (100)	1 (100)	–	1 (100)	1 (100)	–	1 (100)
	I	–	–	1 (100)	–	–	–	–	–	–	–	–	–
	R	1 (100)	–	–	–	–	–	–	1 (100)	–	–	1 (100)	–
***Acinetobacter*** **spps*. n*** **= 1**	S	–	–	–	1 (100)	1 (100)	1 (100)	1 (100)	1 (100)	–	1 (100)	–	–
	R	1 (100)	1 (100)	1 (100)	–	–	–	–	–	1 (100)	–	1(100)	–
Total *n* = 44	S	–	20(45.5)	5 (11.4)	33 (75)	33 (75)	34(77.3)	43(97.73)	18(40.9)	30(68.18)	33 (75)	8(18.18)	31(70.5)
	I	–	11 (25)	9(20.46)	3 (6.8)	1 (2.3)	3 (6.8)	–	4 (9.1)	5 (11.37)	2 (4.5)	7 (15.9)	3 (6.8)
	R	44(100)	13(29.6)	30(68.2)	8(18.2)	10(22.7)	7 (15.9)	1 (2.27)	22 (50)	9 (20.5)	9(20.5)	29(65.91)	10(22.5)

Note: *Sen.* Sensitivity, *AMP* Ampicillin, *GEN* Gentamycin, *AMC* Augmentin, *CXT* Cefoxitin, *CTX* Cefotaxime, *CIP* Ciprofloxacin, *MER* Meropenem, *COT* Cotrimoxazole, *CAZ* Ceftazidime, *CHL* Chloramphenicol, *TET* Tetracycline, *NIT* Nitrofurantoin, *S* Sensitive, *I* Intermediate, *R* Resistant

**Table 3:** Drug resistant patterns of Gram-positive bacterial isolates from urine samples of children at the FHCSH, Northwest Ethiopia, February–-April, 2019

Bacterial isolate	Sensitivity	ERY	CXT	CPR	TET	CHL	COT	GEN	NIT
*S.aurues*, *n* = 3	S	1(33.33)	2(66.7)	2(66.7)	1(33.33)	2(66.7)	2(66.7)	2(66.7)	3(100)
	I	1(33.33)	–	–	–	1(33.33)	–	–	–
	R	1(33.33)	1(33.33)	1(33.33	2(66.7)	–	1(33.33)	1(33.33)	–
*S.saprophyticus*, *n* = 3	S	2(66.7)	2(66.7)	2(66.7)	–	2(66.7)	2(66.7)	2(66.7)	3(100)
	I	1(33.33)	–	1(33.33)	2(66.7)	1(33.33)	–	–	–
	R	–	1(33.33)	–	1(33.33)	–	1(33.33)	1(33.33)	–
Total, *n* = 6/50	S	3(50)	4(66.67)	4(66.67)	1(16.67)	4(66.67)	4(66.67)	4(66.67)	6(100)
	I	2(33.33)	–	–	2(33.33)	2(33.33)	–	–	–
	R	1(16.67)	2(33.33)	1(16.67)	3(50)	–	2(33.33)	2(33.33)	–

### Antimicrobial susceptibility patterns of gram-negative bacterial isolates

Among tested Gram-negative bacterial isolates, about 43(97.7%) were susceptible to meropenem, followed by ciprofloxacin 34(77.3%), cefoxitin 33(75%), ceftazidime 30(68.2%), and chloramphenicol 33(75%). However, 44(100%) resistance rate was observed to ampicillin followed by augmentin 39(88.6%) and tetracycline 36(81.8%). Above 75% of *E. coli* isolates were susceptible to cefoxitin, cefotaxime, ceftazidime, ciprofloxacin, meropenem, chloramphenicol, and nitrofurantoin. *K. pneumoniae* was another bacterial isolate that showed a high level of susceptibility to meropenem 5/5(100%), ceftazidime, and nitrofurantoin 4/5(80%) each (Table 2).

### Multidrug resistance patterns of bacterial isolates

The overall multidrug resistance rate of the isolates was assessed and about 66% (95% CI 52–78, *N* = 33) showed multidrug resistance. A higher rate of MDR was observed in Gram-negative bacterial isolates compared to Gram-positives. The majority of the Gram-negative isolates 31/44 (70.5%) showed multidrug resistance (MDR). Particularly, the highest MDR was observed in *Klebsiella* specie, 6/7 (85.7%) followed by *Citrobacter* species, 5/6 (83.3%), and *E. coli* species, 18/44 (64.3%) (Table 4).

**Table 4:** Multi-drug resistant profile of Gram-negative bacteria isolated from urine of UTI suspected children attending the FHCSH, Northwest, Ethiopia, February–April, 2019

Antimicrobial resistance pattern	Bacterial isolate					Total
	*E. col*	*Klebsiella* sp	C*itrobacte*	*Acinetobacter*	*Providencia*	
AMP, TET, NIT	1					1
AMP, COT, TET	2				1	3
AMP, GEN, TET	1	1				2
AMP, GEN, AMC	1					1
AMP, AMC, COT	2					2
AMP, GEN, AMC, TET		1		1		2
AMP, AMC, CIP, TET	1					1
AMP, CIP, COT, CHL	1					1
AMP, AMC, COT, TET	1	1				2
AMP, GEN, AMC, CTX, TET	1					1
AMP, GEN, COT, TET, NIT	1					1
AMP, AMC, COT, CHL, TET		2				2
AMP, GEN, AMC, CHL, NIT	1					1
AMP, AMC, CTX, CIP, COT, TET	1					1
AMP, GEN, AMC, CXT, CTX, COT, CAZ			1			1
AMP, GEN, AMC, COT, CAZ, CHL, TET	1					1
AMP, AMC, CXT, CTX, COT, CHL, TET, NIT			3			3
AMP, AMC, CXT, CTX, COT, CAZ, CHL, TET			1			1
AMP, AMC, CXT, CIP, COT, CAZ, TET, NIT	1					1
AMP, GEN, AMC, CTX, CIP, COT, CAZ, TET		1				1
AMP, GEN, AMC, CXT, CTX, CIP, COT, CAZ, CHL, TET	1					1
AMP, GEN, AMC, CXT, CTX, CIP, MER, COT, CAZ, CHL, TET	1					1
Total	18	6	5	1	1	31

### Extended spectrum beta-lactamase production

In the beginning, a total of seven Gram-negative bacterial isolates (**six** *E. coli* and one *K. pneumoniae*) were candidates for ESBL production test. 6 bacterial isolates (5 *E. coli* and **1** *K. pneumoniae*) were phenotypically confirmed for the production of ESBL by using the double disk diffusion method; ceftazidime [30 μg] and ceftazidime-clavulanic acid [30/10 μg]. A difference of ≥5 mm between zone diameters of ceftazidime and ceftazidime-clavulanic acid disk is taken to be phenotypic confirmation of ESBL production with fulfilling the criteria of CLSI-2018 [23].

### Antimicrobial susceptibility patterns of gram-positive bacterial isolates

All Gram-positive isolates were 6/6(100%) sensitive to nitrofurantoin followed by cotrimoxazole, cefoxitin, ciprofloxacin, chloramphenicol, and gentamycin, 2/3(66.7%) each. About 1/3(33.33%) of *S. aureus* showed MDR.

### Associated risk factors

Based on the multivariate logistic regression model, two factors, such as the circumcision status of males (AOR = 18.99, 95% C.I. = 5.5–65.353) and history of UTI (AOR = 6.42, 95% CI = 1.86–22.152) were found to be the independent risk factors for UTI in children. Therefore, being previously infected with UTI had more than six times the likelihood of developing UTIs compared to those who had not infection. On the other hand, males who were not circumcised were 19 times higher to be culture positive than circumcised boys (Table 5).

**Table 5:** Predictors of urinary tract infections among children who attend pediatric OPD of FHCSH, Northwest Ethiopia, February to April, 2019

Variables		UTI N (%)	No. UTI (%)	***P***-value	COR	***P***-value	AOR
**Age**	< 1	11 (3.7)	25 (8.4)	0.043	0.372 (0.143_0.962)	0.237	0.408(0.092_1.804)
	1_5	10 (3.3)	95 (31.8)	0.067	2.117 (0.949_4.723)	0.111	3.12 (0.766_13.36)
	6_15	29 (9.7)	129 (43.1)		1		1
Sex	Female	30 (10)	145 (48.5)	0.022	2.07 (1.11_3.86)		
	Male	20 (6.7)	122 (73.9)		1		
Circumcision in boys	No	15 (9.1)	23 (13.9)	0.00	15.93 (5.27_48.09)	0.00	18.99 (5.5_65.35) *
	Yes	5 (3.0)	104 (34.8)		1		1
Residence	Rural	10 (3.3)	81 (27.1)	0.08	1.98 (0.92_4.28)		
	Urban	40 (13.4)	168 (56.2)		1		
Diabetes mellitus	Yes	13 (4.3)	16 (5.4)	0.00	0.195 (0.09_0.44)		
	No	37 (12.4)	233 (77.9)		1		
History of UTI	Yes	31 (10.4)	19 (6.4)	0.00	0.09 (0.05_0.18)	0.003	6.42 (1.86_22.15) *
	No	32 (10.7)	217 (72.6)		1		1
History of catheterization	Yes	31 (10.4)	19 (6.4)	0.00	0.06 (0.03_0.13)		
	No	23 (7.7)	226 (75.6)		1		
Education of father	Illiterate	19 (3.0)	74 (24.7)	0.14	2.01 (0.79_5.14)		
	primary	17 (5.7)	74 (24.7)	0.88	1.07 (0.47_2.43)		
	High school	12 (4.0)	52 (17.4)	0.89	1.07 (0.47_2.58)		
	College and above	12 (4.0)	49 (16.4)		1		
Education of mother	Illiterate	18 (6.0)	109 (36.5)	0.48	0.69 (0.24_1.97)		
	Primary	11 (3.7)	54 (18.1)	0.311	0.56 (0.18_1.73)		
	High shcool	16 (5.4)	42 (14.0)	0.03	0.30 (0.10_0.0.89)		
	College and above	5 (1.7)	44 (14.7)		1		
Malnutrition	Yes	18(6)	32(10.7)	0.00	3.81(1.92_7.58)		
	No	32(10.7)	217(72.6)		1		

## Discussion

The prevalence of UTI in the present study was 16.7% (95% CI 12.7–20.4, *N* = 50). It is in line with the study done in Addis Ababa 15.8% [18]. However, it is lower than the findings of the studies in Gondar, 26.45% [19], and Hawassa, 27.5% [8]. This difference might be a female predominance to Gondar University Hospital, 60.94% in contrast to our study 44.8%. The majority of male study participants, 71.7% in the Hawassa University Hospital were not circumcised. Therefore, a tight foreskin may interfere with the normal passage of urine and can prevent the full emptying of the bladder [25, 26].

Though UTI can be caused by both Gram-negative and Gram-positive bacteria, Gram-negative bacteria are the most common cause of the infection, because the agents are the normal constituent of the normal intestinal microbiota [27]. Acquisition of UTI starts with periurethral contamination by uropathogens inhabiting in the gut, followed by colonization of the urethra and successive migration of the pathogen to the bladder [28]. Among these, *E. coli* is the predominant one [14, 29, 30]. Similarly, the present study reveals that 88% (44/50, 95% CI 80–96%) of the isolate are Gram-negative bacteria. Of these, *E. coli* accounted for about 63.63% of Gram-negatives followed by *Klebsiella* species (15.9%), and *Citrobacter* species (13.63%). This predominance might be due to their unique structures such as flagella and pili, which help for their attachment to the uroepithelium and increases risks for infection [31]. Similar percentage of *Citrobacter species, E. coli* species, and *Klebsiella* species was documented in Nigeria, 10.7% [32], in Iran, 57.4% [33], and in Scotland, 19% [3] respectively. A study in Bangladesh reported the same prevalence of *E. coli*, 63.3% of the total Gram-negative bacterial isolates [34]. About 30/50 (60%) of the isolate and 20/28 (72%) of *E. coli* and *Klebsiella* species were isolated from females. This might be due to poor hygienic conditions, the proximity of anal and urethral openings, and relatively wide urethra [14].

All Gram-negative bacterial isolates 44/44(100%) were resistant to ampicillin followed by augmentin 39/44(88.6%) and tetracycline 36/44 (81.8%). Similar findings were seen in Scotland, 93% [35] and in Pakistan, 100% [36]. The reason may be due to the continuous use of these drugs for many years, easily availability, self-prescription, the tendency of patients using relatively cheaper antibiotics for all types of infection, and misuse. About 43/44(97.3%) of isolates were sensitive to Meropenem. This might be due to the unavailability of this drug in the area. As Table 2 shows, ciprofloxacin, cefoxitin, and ceftazidime showed the best performance against *E. coli*. This finding was almost similar with the findings reported at Gondar Hospital and Yekatit 12 hospital [18]. According to the findings in Gondar Hospital, the sensitivity of gentamicin and cotrimoxazole to *E. coli* was 80 and 73% [19] respectively. However, in the present study, the susceptibility to the above-listed drugs were 15/28(53.4%) and 16/28(57.1%) respectively. This might be due to the gradual increase of drug resistance/selective pressure of bacteria to the drug /mutation, or the difference in antibiotic practices in the study area. Similar research from Iraq also revealed 75% sensitivity to chloramphenicol [2].

All Gram-positive isolates showed 6/6(100%) sensitivity to nitrofurantoin. A similar result was reported at Yekatit 12 hospital 100% [18].The reason for the effectiveness of this drug might be, due to the nature of having multiple mechanisms and site of actions of the drug with a non-specific blockage of protein synthesis limited access to the drug, and narrow-spectrum nature of the drug. However, about 71.4% resistance rate was documented in Hawasa [8]. Higher resistance to this antibiotic is perhaps due to its wrongly usage as an empirical therapy.

Furthermore, the overall MDR prevalence in this study was (66%) (95% CI 52–78, *N* = 33). Specifically, Gram-negative bacterial isolates showed significant level of MDR 31/44(70.5%) compared to Gram-positive bacteria 2/6(33.33%). This was comparable to the findings reported in Gondar 58.53% [30] and Addis Ababa, 73.7% [18]. Although antimicrobial resistance comes primarily as a result of selective pressure on susceptible microbes by the use of therapeutic agents, there are also further multiple factors for the spread of resistance. Using broad-spectrum agents, easy availability of antimicrobials in non-controlled pharmacy, sub-standard/poor drug quality, treatment termination, and over-prescription due to a poor diagnostic set-up or fear of loss of follow-up are among common factors enhancing antimicrobial resistance [37].

The emergence of resistant strains among uropathogens are alarmingly increasing with different resistance patterns [38]. Acquisition of resistance might be either mutational (changing the target site of a bacteria within its genetic material) or acquisition of new genetic material from other bacteria. This problem is also magnified by an irrational use and poor administration of drugs. Once a patient acquires resistant strain bacteria, then it transfers antibiotic resistance genes to other bacteria [39].

The present study also demonstrated the prevalence of ESBL producing strains which was 13.6% (95% CI 6.5–32.3%, *N* = 6). The prevalence is dynamically rising just due to multiple factors; lack of early treatment of the case, fast transmission ESBL strains to the community and healthcare institutions, and environmental stress. Inappropriate use of carbapenems, increased use of third-generation cephalosporin and quinolones in the community, stool-mediated infections, and the existence of the ESBL gene in the plasmid are other possible factors [40]. Having plasmids and other mobile genetic materials can help ESBL producing bacterial strains to be resistant for other multiple antimicrobial agents. This may let physicians to have narrow treatment options and increase the mortality and morbidity of patients infected with ESBL producing bacteria [41].

In this study, associated factors were also determined. Among assessed factors, the history of previous UTI and male uncircumcision was the independent risk factors for the acquisition of UTI. A recurrent urinary tract infections can be attributed to two common enhancing factors: ascending of identical urethral microbiota, particularly uropathogenic *E. coli* to the bladder and/or untreated chronic/persistent bladder infection resulted from either ascending or bloodstream infections [42]. This fact is supported by a study done in Denmark, and it depicted that 77% of recurrent UTIs have resulted from infection with identical uropathogenic *E. coli* strains [43]. Adhesion structures like P and type 1 pili may help *E. coli* for progression to the bladder [44].

Having facultative intracellular multiplication in uroepithelial cells helps *E. coli* to escape from being killed by humoral immunity and antimicrobial agents. This multiplication takes place to a certain extent and forms loose colonies and escapes out to the lumen of the bladder. However, some of the bacterial colonies remain intracellularly and can be a reservoir for persistent infection [45]. This study, however, couldn’t assess the molecular characteristics of bacterial isolates.

## Conclusion

The prevalence of UTI in children was high, and considerably a high proportion of MDR strains were observed in the present study. Previous history of UTI and male uncircumcision were the predictor variables of UTI.

## Data Availability

The datasets used and/or analyzed during the current study are available from the corresponding author on reasonable request.

## Abbreviations

AOR: Adjusted Odds Ratio
ATCC: American Type Culture Collection
CI: Confidence Interval
CLED: Cysteine-Lactose-Electrolyte Deficient Agar
CLSI: Clinical Laboratory Standards Institute
CSA: Central statistical agency of Ethiopia
ESBL: Extended Spectrum Beta Lactamase
FHCSH: Felege-Hiwot Comprehensive Specialized Hospital
MDR: Multi-Drug Resistance
MHA: Muller-Hinton Agar
MSU: Mid-Stream Urine
UTI: Urinary Tract Infection
